# Path4Drug: Data Science Workflow for Identification of Tissue-Specific Biological Pathways Modulated by Toxic Drugs

**DOI:** 10.3389/fphar.2021.708296

**Published:** 2021-10-14

**Authors:** Barbara Füzi, Jana Gurinova, Henning Hermjakob, Gerhard F. Ecker, Rahuman Sheriff

**Affiliations:** ^1^ Department of Pharmaceutical Sciences, University of Vienna, Vienna, Austria; ^2^ European Molecular Biology Laboratory, European Bioinformatics Institute, Hinxton, United Kingdom; ^3^ Beijing Institute of Lifeomics, National Center for Protein Sciences, Beijing, China

**Keywords:** toxicicity, drugs, drug targets, biological pathways, data science

## Abstract

The early prediction of drug adverse effects is of great interest to pharmaceutical research, as toxicity is one of the leading reasons for drug attrition. Understanding the cell signaling and regulatory pathways affected by a drug candidate is crucial to the study of drug toxicity. In this study, we present a computational technique that employs the propagation of drug-protein interactions to connect compounds to biological pathways. Target profiles for drugs were built by retrieving drug target proteins from public repositories such as ChEMBL, DrugBank, IUPHAR, PharmGKB, and TTD. Subsequent enrichment test of the protein pool using Reactome revealed potential pathways affected by the drugs. Furthermore, an optional tissue filter utilizing the Human Protein Atlas was applied to identify tissue-specific pathways. The analysis pipeline was implemented in an open-source KNIME workflow called Path4Drug to allow automated data retrieval and reconstruction for any given drug present in ChEMBL. The pipeline was applied to withdrawn drugs and cardio- and hepatotoxic drugs with black box warnings to identify biochemical pathways they affect and to find pathways that can be potentially connected to the toxic events. To complement this approach, drugs used in cardiac therapy without any record of toxicity were also analyzed. The results provide already known associations as well as a large amount of additional potential connections. Consequently, our approach can link drugs to biological pathways by leveraging big data available in public resources. The developed tool is openly available and modifiable to support other systems biology analyses.

## Introduction

The interaction between a chemical compound and the human body can be a complex cascade of different mechanisms. For understanding these mechanisms, the use of systems biology approaches, which are moving away from the one drug-one target concept, has seen a consequent rise in the last couple of years. Biological pathways are described as a sequence of interactions between biochemical molecules. Systems biology approaches such as pathway-level analysis of drug actions can reveal information about the underlying mechanisms induced by the drug. Furthermore, it can help to increase our understanding of the possible adverse events in connection to the compound (Yahya et al., 2021).

The National Research Council described the concept of toxicity pathways as a regular biological pathway that becomes perturbed beyond the normal homeostasis of the organism leading to toxicity (Council National Research, Division on Earth and Life Studies, 2007). On this basis, existing biological pathways could be connected to toxic events. Defining pathways involved in such events can help set a warning sign towards drugs that affect certain pathways (Ganter and Giroux 2008). As an example, selective COX-2 inhibitors (celecoxib, rofecoxib, valdecoxib) received a warning from the FDA because the pathways they target are related to the mechanism of cardiovascular toxicity, and shortly afterward, they were withdrawn from the market (Yuryev 2008). Therefore, pathway-based toxicology is an important field, providing tools for interpreting and understanding the increasing amount of toxicity data (Hartung et al., 2017). Analyzing toxic compounds and pathway relations provides a way to shed light on pathways potentially involved in toxic mechanisms.

Drugs usually affect multiple biological pathways. To understand the toxicological relevance of pathways affected by toxic drugs, drug-pathway connection data is needed. There are databases available capturing biological pathways: Reactome (Jassal et al., 2020), KEGG (Kanehisa et al., 2017), and Wikipathways (Slenter et al., 2018). Some of them contain compound relations as well. However, these connections are often limited to drug metabolism or mode of action pathways (Zeng et al., 2015). Therefore, they are most probably unable to capture a complete picture of the relations between drugs and biological pathways.

The aim was to connect drugs to biological pathways through proteins they modify to obtain a complete overview of the drug’s systemic effect. Our approach uses broader drug-target profiles for uncovering unknown connections between drugs and biological pathways and eventually between toxic events and pathways, using statistical analysis.

In the first instance, the focus was on drugs which have been withdrawn because of their toxic properties and black box warning cardio- and hepatotoxic compounds. Withdrawn drugs were once approved and later withdrawn from the market for toxicity or efficacy reasons. The FDA has introduced black box warnings to call attention to serious or life-threatening risks. With our method, we aimed to place cardio- and hepatotoxic events into their biological context and to gain more knowledge on two of the most often occurring organ-specific toxicities. A tissue-specific filtering system for the collected target proteins was also included to discover the organ level events’ characteristics. By completing an overrepresentation test with the retrieved tissue-specific target profiles, our aim was to define pathways relevant to the toxicity classes. We developed Path4Drug, an open-source KNIME workflow, which allows the analysis of other datasets as well, to support other pathway-oriented systems biology approaches.

## Materials and Methods

### Path4Drug Input Data Selection

The input of the workflow is a drug or compound, and it should be provided as a ChEMBL identifier or a list of identifiers for multiple compounds. When a list compound IDs is provided, the workflow can sequentially run for each compound.

Our analysis pipeline was based on a set of cardiotoxic and hepatotoxic compounds for identifying target proteins and biological pathways that can be involved specifically in one type of organ-level toxicity. The compounds were selected from drugs labeled as withdrawn or black box warning compounds by ChEMBL (Hunter et al., 2021).

First, drugs from four of the most common organ or organ system-specific toxicity classes across withdrawn and black box warning drugs–neuro-, nephro-, cardio- and liver toxicity - were extracted. Since the main aim comprised a comparison of toxicity classes, compounds that are present in more than one of the classes and therefore cannot be explicitly connected to one toxicity type were excluded. Thus, there is no overlap between the compound groups. The filtered list contained 57 cardio, 75 hepato-, 12 nephro-, and 28 neurotoxic compounds. The subsequent analysis was carried out with the two most populated classes, i.e., cardio- and hepatotoxic compounds.

Additional compounds with cardiovascular toxicity from the withdrawn database were used as an external test set (Siramshetty et al., 2016). Since the number of withdrawn compounds is limited, we could only find four compounds with target data, which were not in our initial dataset (see Supplementary Material.) Nevertheless, we applied the analysis workflow to this group, to confirm the results of the initial cardiotoxic group.

Furthermore, the workflow was also applied on approved cardiac therapy drugs that are still on the market and are not connected to toxicity, to compare the pathways they affect with those that are modulated by cardiotoxic compounds. Small molecules from the category “cardiac therapy” from ChEMBL were downloaded with a maximum phase of 4, which means they were approved. The already withdrawn ones and the ones with a black box warning were filtered out from the cardiac therapy list. The final set contains 27 compounds.

Consequently, the cardiotoxic compounds had comparison groups, allowing the distinction of pathways between two tissue-specific toxicity classes and between therapy and toxic compounds of the same tissue-group, allowing identification of pathways that could play a specific role in cardiotoxicity.

### Path4Drug KNIME Workflow

**FIGURE 1:**
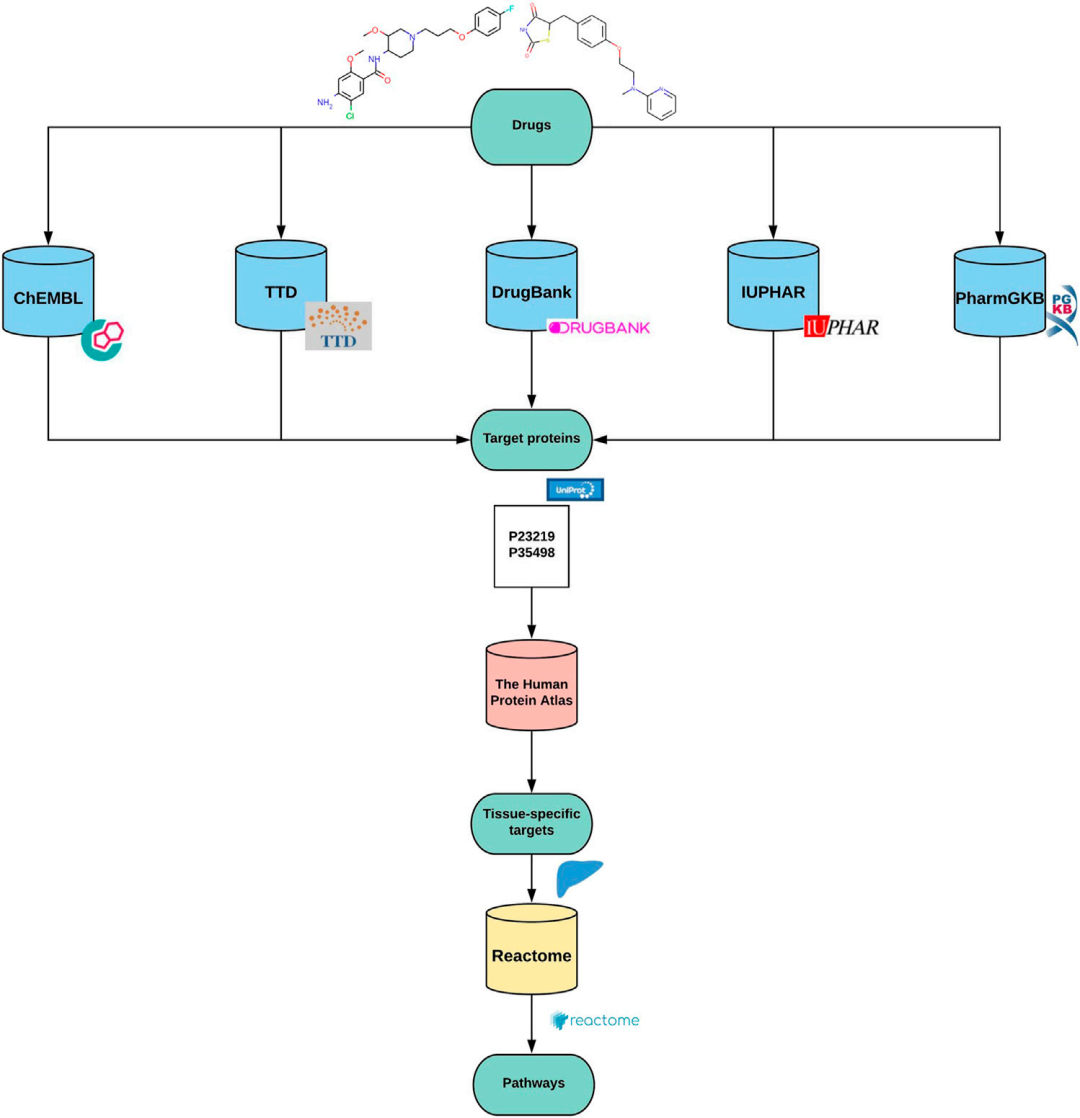
Graphical overview of the method.

Path4Drug is a computational pipeline that employs drug-protein interactions to identify biological pathways perturbed by a drug. Path4Drug was implemented as a KNIME workflow (version: KNIME 4.1.2) to perform automated data retrieval and perturbed pathway prediction. KNIME is an open-source, graphical-analytics platform, and workflow management system, created for data science purposes (Berthold et al., 2009). Figure 1 outlines the graphical overview of the approach. The steps involved in the workflow are described below.

Step 1 Building protein target profiles

In this step, for each inputted drug, a protein-target profile was built by collecting target proteins from five different databases: ChEMBL (version 27) (Mendez et al., 2019), DrugBank (version 5.1.6) (Wishart et al., 2018), IUPHAR (version 2020.4) (Armstrong et al., 2020), TTD (last updated June 1^st^, 2020) (Wang et al., 2020), and PharmGKB (downloaded files: 09.2020) (Whirl-Carrillo et al., 2012). The target data in these databases are curated by scientific experts, hence are reliable and comparable. We defined targets as proteins, for which a drug was labeled as active in a biological assay or was annotated as a proven target for the drug in one of the queried databases. These target databases differ in their architecture and accessibility. Thus, we applied different approaches as described below to retrieve and prepare the data for further usage in the workflow. Although all these databases are open for academic use, their license policy varies. Hence users of our workflow should ensure they have the necessary license to use them or modify the workflow accordingly to amend the list of databases used for target collection.

#### ChEMBL

ChEMBL (https://www.ebi.ac.uk/chembl) is a database of bioactive molecules with drug-like properties, manually curated from public literature. The web services offered by ChEMBL (Davies et al., 2015) were used in the workflow to extract drug target protein data. At first, for each inputted drug, an assay search was performed across the database to query all proteins showing bioactivity value of pChEMBL 5 or above. pChEMBL is defined as: −log_10_ (molar IC50, XC50, EC50, AC50, Ki, Kd or Potency) (Bento et al., 2014). After querying as described above, all available target proteins were grouped into human and non-human proteins.

With the human targets, a second ChEMBL API call was performed to gain more information on the targets, such as the UniProt accession number. We were interested in protein-type targets, therefore, other types of targets were discarded from the analysis. In case the target type was a whole protein family, the accession numbers of all members were added to the protein pool. UniProt contains reviewed and unreviewed entries. Only the manually curated, reviewed ones were considered in our analysis.

From the non-human targets, the non-mammalian ones were eliminated by using the organisms ChEMBL API call. Based on the hypothesis that mammalian targets act similarly to human ones, we intended to enrich the sparse human data with translated mammalian targets. This part of the workflow can be viewed as optional considering the inconsistent concordance rate of translational studies (Leenaars et al., 2019). However, ignoring the non-human proteins will make the available data more limited and therefore the scope of the analysis narrower. Since the gene symbol is identical across different species, the human equivalents of the mammalian targets were queried and added to the list as well by using the Proteins API provided by the EBI protein service (Nightingale et al., 2017).

In addition to the above approach, ChEMBL’s mode of action API call was used to retrieve proven therapeutic targets. After accessing and filtering the mechanism of action target data, the received targets were also converted into UniProt accession numbers. As a result of both approaches, for each drug queried a collection of target proteins with UniProt accession numbers were extracted from ChEMBL.

#### DrugBank

The DrugBank (https://go.drugbank.com) pharmaceutical knowledgebase provides a download option for non-commercial usage of the openly available portion of their data in the form of one XML file. From that file, the target information was extracted for further processing. Since the processing of the whole XML is excessive, an excel file with the information needed was created and read into the workflow for further usage. Since the whole workflow starts with a ChEMBL identifier or a list of CHEMBL identifiers, the UniChem service (Chambers et al., 2013) was utilized to map DrugBank and ChEMBL IDs to allow the assignment of the target information to the appropriate compounds.

#### Therapeutic Target Database

Drug target protein dataset was downloaded from the Therapeutic Target Database (TTD) (http://db.idrblab.net/ttd/). The drug identifier cross-matching and the drug target mapping files provide information for building the target profiles. Neither does the drug ID cross-matching include ChEMBL identifiers, nor does the UniChem translation service support TTD. Hence PubChem compound IDs were chosen as a base for mapping, as these identifiers are present in both sources. As the drug and target file has no UniProt accession numbers, a python script provided by UniProt was modified and introduced to the workflow for translating the UniProt names to their accession numbers. The output is a list of ChEMBL IDs with their targets by UniProt accession numbers.

#### Pharmacogenomics Knowledge Base

Similar to TTD, compound mapping and relationship files were downloaded from the Pharmacogenomics Knowledgebase (PharmGKB) database (https://www.pharmgkb.org). As PharmGKB IDs are supported by UniChem, a direct translation to ChEMBL IDs was feasible. From the relationship files, rows that symbolize a relationship between a drug and a target gene were extracted. The UniProt accession numbers of reviewed human proteins expressed by the target genes were queried via the EBI Protein API.

#### Guide for Pharmacology

IUPHAR (Guide for Pharmacology) (https://www.guidetopharmacology.org) offers web services for protein target search. The ChEMBL IDs were translated to IUPHAR IDs and used in the IUPHAR’s ligand-interactors API, which returned JSON file with target data. IUPHAR has one identifier for a protein regardless of the organism; for instance, Catechol-O-methyltransferase with the target ID 2472 represents the human, mouse, and rat proteins. With the EBI Protein ID mapping followed by filtering steps the human equivalent UniProt accession numbers of each target entry were obtained.

Step 2 Unifying the list of protein targets

The collected drug-protein pairs from each database were harmonized and collated, resulting in a list with ChEMBL ID for drugs and UniProt accession number for target proteins. It was ensured, that the final list only contains reviewed human proteins, and the duplicate drug-protein pairs were removed. After slimming the data to unique pairs, a protein pool was built for every compound by concatenating the target proteins separately for each drug`s ChEMBL ID.

Step 3 Tissue-specific target profiles

Subsequently, an optional filtering, which removes protein targets not expressed in a tissue, was added to the workflow to support the analysis of a specific tissue type. The expression data from The Tissue Atlas of The Human Protein Atlas (https://www.proteinatlas.org); (Uhlén et al., 2015) was used to build this filter. The Tissue Atlas contained expression data of mRNA and protein levels. Since the protein expression was not available for some genes (for instance the hERG potassium channel), the consensus mRNA expression was chosen, which is a merged dataset from three sources. The tissue of interest was selected, and genes with low or no expression in that tissue were removed from the drug target profiles. As we focused on cardio- and hepatotoxicity, we built target profiles for the drug groups with sub-list of proteins that are expressed in the heart and the liver, respectively.

Step 4 Connecting drugs and biological pathways

With the list of tissue-specific target proteins for each drug, a statistical overrepresentation test was performed through the Reactome pathway analysis API service (Fabregat et al., 2017). Reactome is a manually curated open-source pathway database, which provides a high-performance pathway analysis service (Jassal et al., 2020).

This statistical (hypergeometric distribution) test analyses whether certain pathways are overrepresented in the submitted data. Overrepresentation is described as the presence of more proteins for a pathway in the submitted list than it would be expected only by chance. The result of this test is a probability score. This probability score is expressed as a p-value and is corrected via the False-discovery rate (FDR) (Benjamini and Hochberg 1995). The smaller the p-value, the higher the significance of the pathway (Kapitulnik 2004). The False-discovery Rate indicates whether the so-called significant features are indeed significant or not. We used a cut-off of 0.05 FDR to select pathways significantly connected to the drug.

The test is completed via a POST request in Reactome. Our request was carried out with a taxonomy identifier of 9606, whereby disease pathways were not included. The request body is equal to the protein pool we built in the first part of the workflow. The JSON file returned from the API call was processed to export the workflow`s a table containing the drug(s), associated pathways and statistical values (Table1).

Additionally, another POST request can be performed by the workflow to retrieve the token of the analysis. With the token and preparation of a JAVA snippet for URL encoding, a GET request can be used to receive a link to the Reactome site´s analysis page or to download the detailed analysis report as a pdf.

**FIGURE 2:**
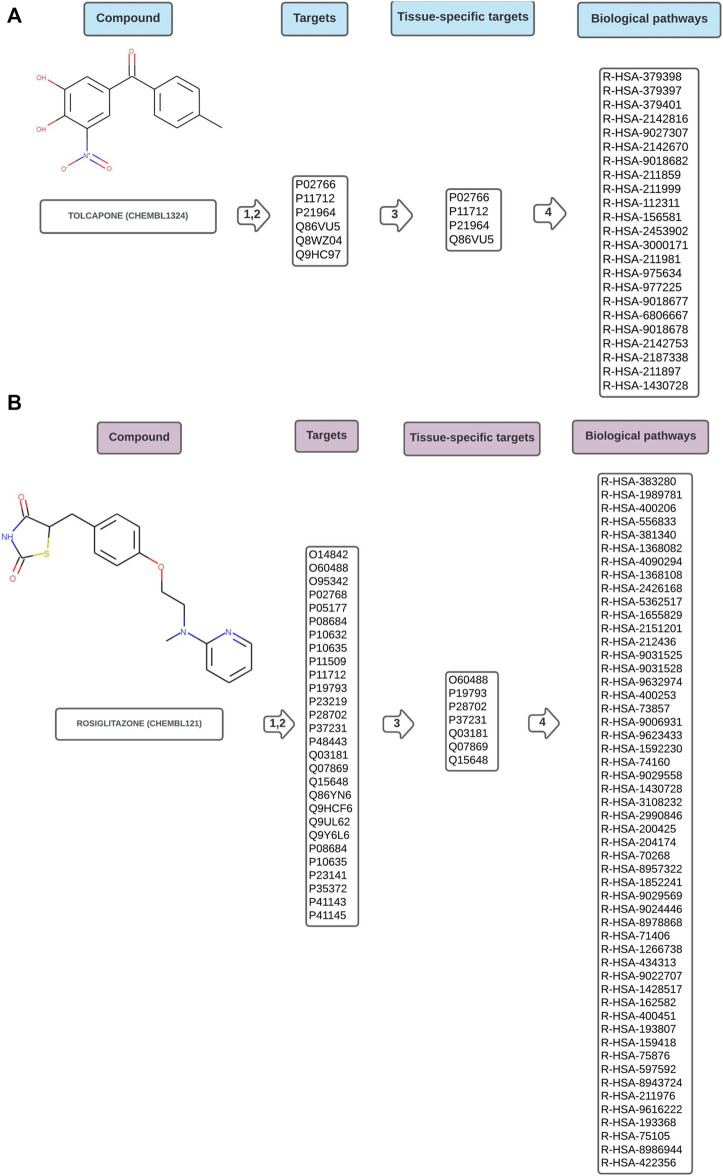
Schematic representation of data flow in Path4Drug KNIME workflow for **(A)** hepatotoxic drug tolcapone (CHEMBL1234, https://www.ebi.ac.uk/chembl/compound_report_card/CHEMBL1324/) and **(B)** cardiotoxic drug rosiglitazone (CHEMBL121, https://www.ebi.ac.uk/chembl/compound_report_card/CHEMBL121/) with the harmonized target list represented by UniProt IDs, the tissue-specific sub-list after filtering, and the biological pathways connected to the compound symbolized by Reactome IDs. As described in the methods section, in step 1,2 the targets were obtained from five different databases and harmonized, in step 3 a tissue-specific filtering was applied and in step 4 pathway overrepresentation test was performed using Reactome.

Figures 2A,B are schematic representations of the data obtained by the workflow, illustrated with one example drug of each toxicity class. The complete result for every analysed compound is available in the Supplementary Material. Supplementary csv Files “hepatotoxic_pathways”, “cardiotoxic_pathways”, “cardiac_therapy_pathways” contain the raw results obtained directly from the worflow. They consist of the ChEMBL identifier for the compound, the concatenated, tissue-specific target list, the name of the pathways, FDRs and pValues, and the Reactome identifiers of the pathways.

**TABLE 1:** Example of the workflow output for cardiotoxic compound CHEMBL1729 (Cisapride) showing top 5 pathways. The output table consist of the compound identifier, pathway names, pathway identifiers, and statistical values such as fdrs and pValue for all significantly modulated pathways.

molecule_chembl_id	Names	stIds	fdrs	pValues
CHEMBL1729	Phase 3 - rapid repolarisation	R-HSA-5576890	0.0037	0.0006
CHEMBL1729	Voltage gated Potassium channels	R-HSA-1296072	0.0090	0.0030
CHEMBL1729	Potassium Channels	R-HSA-1296071	0.0097	0.0073
CHEMBL1729	Cardiac conduction	R-HSA-5576891	0.0097	0.0097
CHEMBL1729	Muscle contraction	R-HSA-397014	0.0152	0.0152
CHEMBL1729	Neuronal System	R-HSA-112316	0.0340	0.0340

### Compound-Pathway Data in Reactome

Reactome (https://reactome.org) is also a repository for downloadable pathway-related data. The data contain ChEBI (Chemical Entities of Biological Interest) compound relations to pathways based on compound-protein interactions. ChEBI (https://www.ebi.ac.uk/chebi/) is a dictionary of molecular entities focused on small chemical compounds (Hastings et al., 2015). We filtered out the non-human pathways from the Reactome data, translated the ChEBI IDs to CHEMBL IDs, and used this data to see if novel connections were detected by our workflow.

## Results

With our analysis of the three compound groups, we intended to highlight differences between pathways that are modulated by cardiotoxic, hepatotoxic, and cardiac therapy compounds, to characterize pathways that are specific for the cardiotoxic group. Furthermore, the results derived by our workflow were compared with compound-pathway connections based on compound-protein interactions annotated by Reactome. In this first general evaluation, we could see that the workflow was able to find most of the connections presented by Reactome and many novel connections. Additionally, the workflow uncovered pathway connections for several compounds, which are not yet present in the Reactome data.

### Comparison of Toxicity Classes

The results of cardiotoxic and hepatotoxic compounds were compared at target, as well as pathway level to identify toxicity specific targets and pathways.

#### Target Profile Comparison of Cardio-and Hepatotoxic Drugs

After building the target profile and applying the tissue filtering, tissue-specific target proteins were found for 54 hepatotoxic and 41 cardiotoxic compounds. 145 proteins were connected to the hepatotoxic and 68 to the cardiotoxic compounds. As expected, based on the different expression patterns of the two tissues, there are only a limited number of shared target proteins. The most relevant targets connected to the two toxicity classes were identified by finding the most often occurring target proteins. Unsurprisingly, many CYP enzymes and the bile salt export pump (ABCB11) are part of the liver pathway list for hepatotoxic compounds, and HERG (KCHN2) is part of the list for cardiotoxic compounds. Proteins, such as Prelamin-A/C (LMNA) are part of both lists (see Supplementary Material).

#### Pathway Profile Comparison of Cardio-and Hepatotoxic Drugs

In Reactome, the pathways are organized hierarchically. For the pathway comparison, the lower-level pathways were considered, which present a more detailed level of the pathway hierarchy. Higher-level pathways such as Cell Cycle or Autophagy are collective terms and were excluded from the analysis because of their generality. An FDR cut-off of 0.05 was applied to filter out less significant pathways. After applying these filtering steps, 382 pathways for 54 hepatotoxic compounds and 420 pathways for 38 cardiotoxic compounds were found. To find the most important pathways, we compared those affected by at least 10% of the cardiotoxic or 10% of the hepatotoxic compounds, and further those affected by at least 20% of the compounds.

**FIGURE 3:**
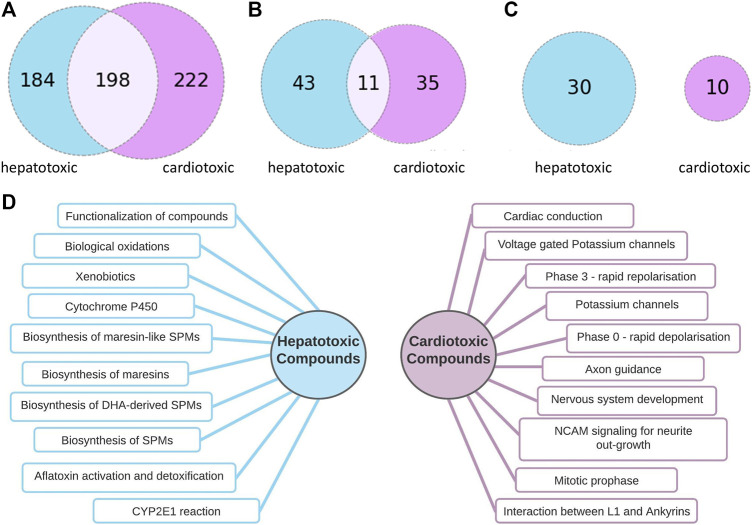
Comparison of pathways connected to hepatotoxic and cardiotoxic drugs **(A)**: Number of pathway connections with an FDR cut-off of 0.05 of the hepatotoxic (blue) and cardiotoxic (purple) group, **(B)**: Number of pathways affected by at least 10% of hepatotoxic (blue) and at least 10 % of cardiotoxic (purple) compounds, 11 common pathways **(C)**: Number of pathways affected by at least 20% of hepatotoxic (blue) and at least 20 % of cardiotoxic (purple) compounds, no common pathways **(D)**: 10 of the most often occurring pathways connected to hepatotoxic (left) and to cardiotoxic (right) compounds.

As Figure 3C implicates, there is no overlap between the most frequently hit pathways by hepatotoxic and the most frequently hit pathways by cardiotoxic compounds. 10 of the most frequently found pathways by each group are summarized in Figure 3D.

### Comparison of Drugs for Cardiac Disorders and Cardiotoxic Drugs

To support our concept of applying this approach for identifying pathways that could play a role in cardiotoxicity, the workflow was in addition run with drugs used to treat cardiac disorders. Having these two groups allowed a comparison between a toxic and non-toxic group connected to the same tissue, supporting the interpretation of the findings of the toxicity group.

#### Target Profile Comparison Between Cardiotoxic Drugs and Therapeutic Drugs for Cardiac Disorders

For 14 drugs for the treatment of cardiac disorders, 34 tissue-specific target proteins were found. 14 of them overlap with the cardiotoxic class, and 20 are exclusive for the cardiac therapy group. The most often occurring targets for the cardiac therapy group are sub-units of the Sodium/potassium-transporting ATPase. The hERG potassium channel, which is a known off-target (Kalyaanamoorthy et al., 2018) is not on the list (see Supplementary Material).

#### Pathway Profile Comparison of Cardiotoxic Drugs and Drugs for Cardiac Disorders

Our goal was to establish which pathways that are connected to cardiotoxic compounds can be specific for the toxicity class. For that purpose, we compared the distribution of pathway connections among the toxic and non-toxic groups.

**FIGURE 4:**
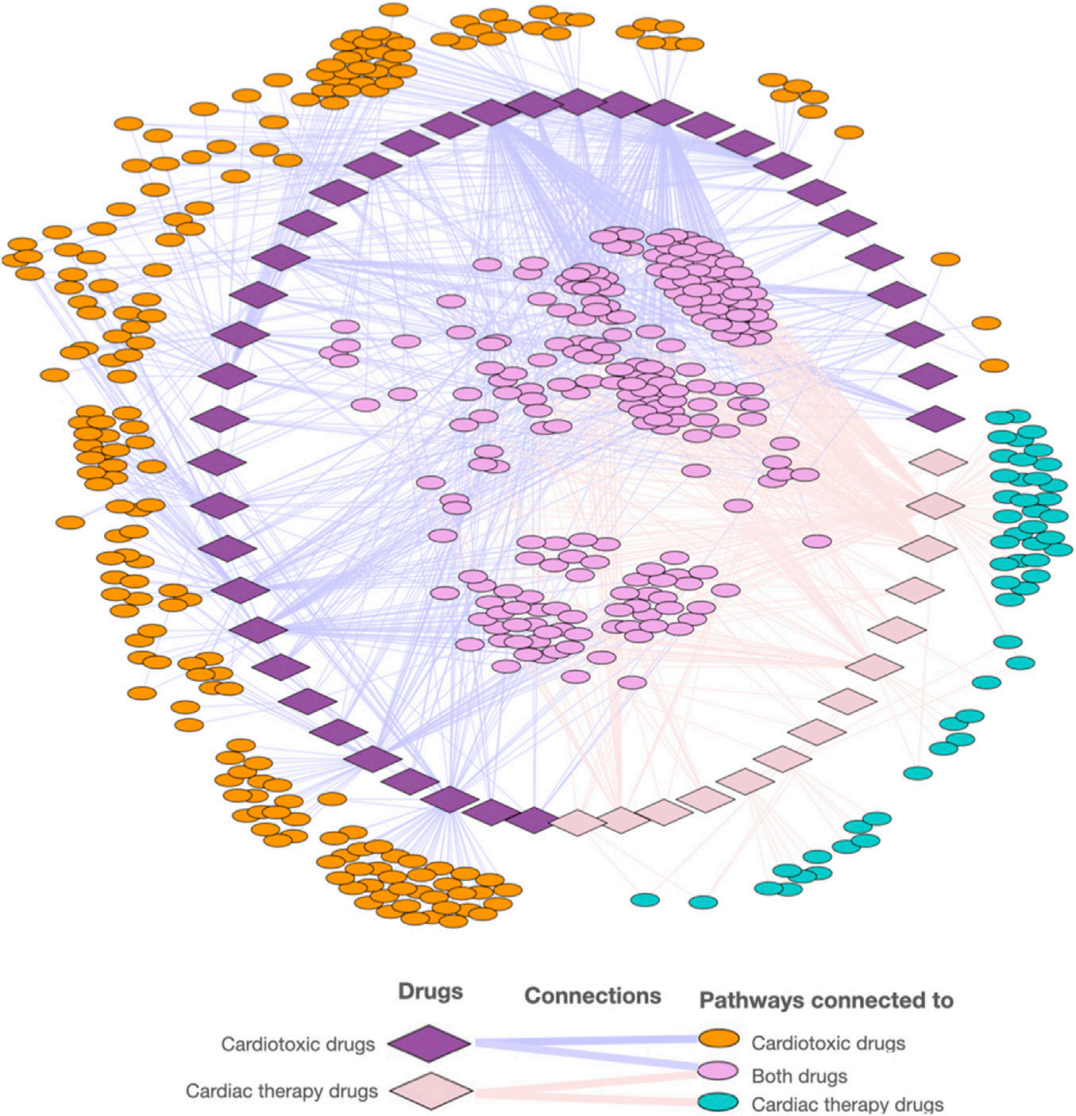
Visualization of the network of cardiotoxic and cardiac therapy compounds and associated pathways.

**FIGURE 5:**
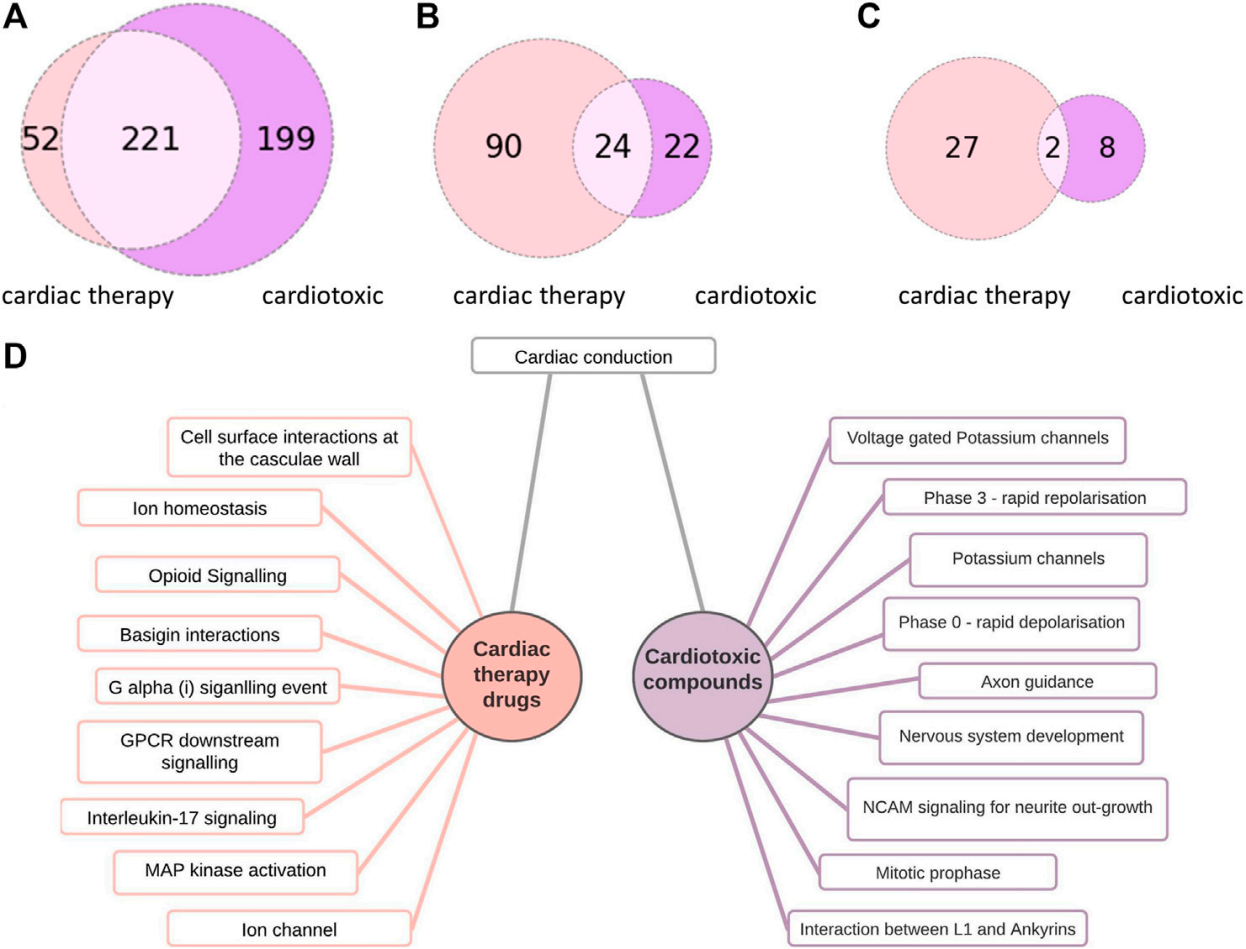
Comparison of pathways connected to cardiac therapy drugs and cardiotoxic drugs **(A)**: Number of pathway connections with an FDR cut-off of 0.05 between the cardiac therapy (light pink) and the cardiotoxic (purple) groups, **(B)**: Number of pathways affected by at least 10% of cardiac therapy (light pink) and at least 10 % of cardiotoxic compounds (purple), 24 common pathways **(C)**: Number of pathways affected by at least 20% of cardiac therapy (light pink) and at least 20 % of the cardiotoxic (purple) compounds, 2 common pathways **(D)**:10 of the most often occurring pathways connected to cardiac therapy drugs (left) and to cardiotoxic drugs (right).

**FIGURE 6:**
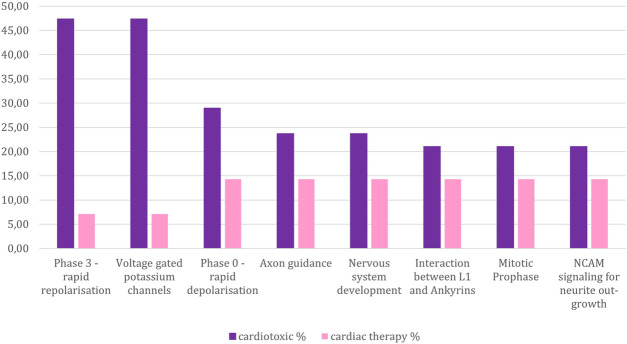
Percentage of compounds from the toxic and non-toxic groups which are associated with those pathways that are connected to at least 20 % of the cardiotoxic compounds, yet less than 20% of the cardiac therapy compounds. Y-axes: compound-percentage, x-axes: pathways illustrated by Reactome terms.

The cardiotoxic drugs can be connected to 420 pathways altogether. 221 of these pathways also can be connected to compounds used to treat cardiac disorders (Figure 4). However, for the pathways connected to at least 10% of the compounds, there are only 24 overlapping pathways (Figure 5B). This number is further reduced to two pathways when refining the list to pathways with a connection to at least 20% of the compounds. From these frequently hit pathways, eight pathways are exclusively connected to cardiotoxic compounds (Figure 5C). The distribution of these eight pathways in the cardiotoxic and cardiac therapy categories is summarized in Figure 6.

## Discussion

A data science pipeline was constructed to connect compounds to pathways by building their tissue-specific target protein profiles and performing an overrepresentation-test with these proteins. The output of the overrepresentation test is a list of pathways that can be connected to the compounds. The application of an FDR cut-off ensured that our list contains only statistically significant pathways. As a use case hepato-and cardiotoxic compounds were analyzed.

In the case of the hepatotoxic compounds, pathways of maresins and other SPMs (specialized proresolving mediators) seem to be frequently affected. SPMs are keys for inflammation determination and for maintaining normal metabolic homeostasis in the liver (Musso et al., 2018). Maresins were already mentioned in connection to the mitigation of liver injuries (Laiglesia et al., 2018; Li et al., 2016). Based on this information, a hypothesis can be built that these pathways could have a crucial role in the organism’s attempt to reduce/avoid hepatotoxic events. Since there are many metabolism pathways modulated, it is plausible that some of the toxic events can be connected to the produced metabolites (Shah et al., 2019).

For cardiotoxic compounds, we compared the list of pathway connections with those of hepatotoxic compounds and of drugs used to treat cardiac disorders. This should allow the estimation if the distinction of cardiotoxic from cardiotonic compounds is possible based on the pathway interaction profiles. This step was completed only for the cardiotoxic category because CHEMBL has the cardiac therapy category in their description list for drugs, making a comparison possible. There is no category across ChEMBL compounds, which can be unambiguously connected to treat liver disorders.

The two pathways namely “Phase 3 - rapid repolarisation”, and “Voltage gated Potassium channels” were most frequently connected to the cardiotoxic compounds, significantly less connected to the cardiac therapy compounds, and not connected to hepatotoxic compounds.: “Phase 3—rapid depolarisation” refers to the third phase of the cardiac action potential, where the potassium ion efflux occurs. The involvement of these two pathways implicates that a change in the action potential causes toxicity by disturbing the potassium ion release. The role of potassium channels in QT prolongation is a known mechanism of cardiotoxicity (Paakkari 2002).

The third most common pathway among the cardiotoxic compounds is “Phase 0—rapid depolarisation”. It is related to the phase of the cardiac action potential, where the influx of sodium ions occurs. For this pathway, the difference of the association to cardiotoxic compounds and cardiac therapy compounds is rather insignificant. This might be, since class I antiarrhythmic agents dominantly affect this part of the cardiac action potential, which comes with the risk of re-entry arrhythmias. Thus, in this group of antiarrhythmic drugs, cardiotoxicity is a frequently observed side effect.

Furthermore, almost every cardiotoxic compound, which can be connected to “Phase 0” can also be connected to “Phase 3”. There are two exceptions, one of them is encainide, which is an antiarrhythmic drug of the Ic class. Ic class antiarrhythmic agents are potent Sodium channel blockers (Pantlin et al., 2020). Encainide was withdrawn from the market due to fatal proarrhythmic side effects (Echt et al., 1991).

There is no non-toxic drug, which can only be connected to “Phase 3” but not to “Phase 0”, which is another indicator for the crucial role of “Phase 3” in cardiotoxicity. Those cardiotoxic compounds that are not connected to “Phase 3”, “Phase 0” or “Voltage gated Potassium channels” pathways (18 compounds altogether) have diverse pathways profiles (see Supplementary Material).

To further verify the significance of these results, compounds with cardiovascular toxicity from the withdrawn database were tested. This test set is small since our dataset and the withdrawn database have considerable overlap in compound information. However, the profile of the four external compounds also implicates the significant involvement of the above discussed pathways, such as “Phase 3—rapid repolarisation” (see Supplementary Material).

Our dataset was relatively small, but the Path4Drug workflow was built with the possibility to apply it to larger sets of drugs and increase the toxicological relevance of the findings. Based on these findings, one could define unfavorable pathway profiles and use them as a warning for possible toxic events. Furthermore, our workflow can be used to find other drug-target-pathway relations, regardless of toxicity to create an overview of the systemic effect of drugs and use this knowledge for instance for drug repurposing. Path4Drug is available on KNIME Hub and modifiable. Consequently, our workflow aims to support pathway-oriented analyses and pathway-driven toxicology approaches.

## Data Availability

The original contributions presented in the study are included in the article/Supplementary Material, further inquiries can be directed to the corresponding authors.
