# Supplementary material for: Path4Drug: Data Science Workflow for Identification of Tissue-Specific Biological Pathways Modulated by Toxic Drugs
Source: Front Pharmacol. 2021 Oct 14;12:708296. doi: 10.3389/fphar.2021.708296 (PMC8551608; doi:10.3389/fphar.2021.708296)
Supplement: Supplementary file 1 [file DataSheet1.ZIP › 708296_supplementary_material_UPDATED/workflow_files/chemicals/README.pdf]

Chemical Download Help

This is an archive of all Chemicals in the PharmGKB knowledgebase. Not all of these chemicals have been involved in PharmGKB annotations.

Multiple "Type" values can be assigned to a given entry since the same substance can be used in different contexts. The "Type" values used for Drugs & Chemicals are as follows:

- Drug = A chemical substance used in the treatment, cure,prevention, or diagnosis of disease.
- Metabolite = Any intermediate or product resulting from metabolism.
- Ion = An atomic or molecular particle having a net electric charge.
- Drug Class = A drug class is a group of medications that may work in the same way, have a similar chemical structure, or are used to treat the same health condition.
- Biological Intermediate = An endogenous small molucule or ion.
- Small Molecule = An electrically neutral entity consisting of more than one atom.
- Prodrug = A compound that must undergo chemical conversion by metabolic processes before becoming the pharmacologically active drug for which it is a prodrug.

Columns in this archive:

1. PharmGKB Accession Id = Identifier assigned to this chemical by PharmGKB
2. Name = Name PharmGKB uses for this chemical
3. Generic Names = Known generic names for this chemical, comma-separated and "-enclosed
4. Trade Names = Known trade names for this chemical, comma-separated and "-enclosed
5. Brand Mixtures = Known brand mixtures this chemical is in, comma-separated and "-enclosed
6. Type = Categories PharmGKB has assigned to this chemical, can be more than one, possible values: Drug, Metabolite, Ion, Drug Class, Biological Intermediate, Small Molecule, Prodrug
7. Cross-references = References to other resources in the form "resource:id", comma-separated
8. SMILES = The SMILES structure for this chemical
9. InChI = The InCHI key for this chemical
10. Dosing Guideline = "Yes" if PharmGKB has annotated a guideline with this chemical, "No" otherwise
11. External Vocabulary = Term for this chemical in another vocabulary in the form "vocabulary:id", comma-separated
