# Supplementary material for: Path4Drug: Data Science Workflow for Identification of Tissue-Specific Biological Pathways Modulated by Toxic Drugs
Source: Front Pharmacol. 2021 Oct 14;12:708296. doi: 10.3389/fphar.2021.708296 (PMC8551608; doi:10.3389/fphar.2021.708296)
Supplement: Supplementary file 1 [file DataSheet1.ZIP › 708296_supplementary_material_UPDATED/workflow_files/genes/README.pdf]

Gene Download Help

This is an archive of all known genes in the PharmGKB knowledgebase. PharmGKB uses HGNC as the source for all its gene records and then assigns each a unique PharmGKB Accession Identifier for use in annotations.

Not all of these genes have been involved in PharmGKB annotations. The best way to filter for PharmGKB-annotated genes is to use the "Has Variant Annotation" field.

1. PharmGKB Accession Id = Identifier assigned to this gene by PharmGKB
2. NCBI Gene ID = Identifier assigned to this gene by NCBI
3. HGNC ID = Identifier assigned to this gene by HGNC
4. Ensembl Id = Identifier assigned to this gene by Ensembl
5. Name = Canonical name for this gene (by HGNC)
6. Symbol = Canonical name for this gene (by HGNC)
7. Alternate Names = Other known names for this gene, comma-separated
8. Alternate Symbols = Other known symbols for this gene, comma-separated
9. Is VIP = "Yes" if PharmGKB has written a VIP annotation for this gene, "No" otherwise
10. Has Variant Annotation = "Yes" if PharmGKB has written at least one variant annotation for this gene, "No" otherwise
11. Cross-references = References to other resources in the form "resource:id", comma-separated
12. Has CPIC Dosing Guideline = "Yes" if PharmGKB has annotated a CPIC guideline for this gene, "No" otherwise
13. Chromosome = The chromosome this gene is on, in the form "chr##"
14. Chromosomal Start = Where this gene starts on the chromosomal sequence
15. Chromosomal Stop = Where this gene stops on the chromosomal sequence

For questions and comments, please contact us at https://www.pharmgkb.org
