# Supplementary material for: Path4Drug: Data Science Workflow for Identification of Tissue-Specific Biological Pathways Modulated by Toxic Drugs
Source: Front Pharmacol. 2021 Oct 14;12:708296. doi: 10.3389/fphar.2021.708296 (PMC8551608; doi:10.3389/fphar.2021.708296)
Supplement: Supplementary file 1 [file DataSheet1.ZIP › 708296_supplementary_material_UPDATED/workflow_files/relationships/README.pdf]

Relationships Help File

Relationship Types:

- ***VariantLocation-Drug*** associations are derived from a VIP summary or a VariantAnnotation or ClinicalAnnotation.
- ***VariantLocation-Disease*** associations are derived from a VIP summary or a VariantAnnotation or ClinicalAnnotation.
    - ***VariantLocation-Disease*** associations derived from a VariantAnnotation or ClinicalAnnotation are really referring to a variant that has an annotation with that "Disease" tag. ***These associations can be misleading because they are not necessarily indications that a variant is directly associated with a disease phenotype.*** For example, in the annotation for rs5275 in PTGS2:
      "Genotype AA is associated with increased progression-free survival and overall survival when treated with capecitabine and oxaliplatin in people with Colorectal Neoplasms as compared to genotypes GG + AG." rs5275 will be listed in the relationships file as "associated" with "Colorectal Neoplasms", but in reality the snp is associated with the treatment outcome for capecitabine and oxaliplatin in people who have the condition.
- ***Haplotype-Drug*** associations are derived from a VIP summary or a VariantAnnotation or ClinicalAnnotation.
- ***Haplotype-Disease*** associations are derived from a VIP summary or a VariantAnnotation or ClinicalAnnotation.
    - ***Haplotype-Disease*** associations derived from a VariantAnnotation or ClinicalAnnotation are really referring to a haplotype that has an annotation with that "Disease" tag. ***These associations can be misleading because they are not necessarily indications that a haplotype is directly associated with a disease phenotype (i.e., disease risk is not implied).***
- ***Gene-Drug*** associations are derived from a PharmGKB pathway, a VIP summary, an FDA drug label, a dosing guideline, or a VariantAnnotation or ClinicalAnnotation.
- ***Gene-Disease*** associations are derived from a VIP summary or from a VariantAnnotation or ClinicalAnnotation.
    - ***Gene-Disease*** associations derived from a VariantAnnotation or ClinicalAnnotation are really referring to a variant in the gene that has an annotation with that "Disease" tag. ***These associations can be misleading because they are not necessarily indications that a gene is directly associated with a disease phenotype.*** For example, in the annotation for rs5275 in PTGS2: "Genotype AA is associated with increased progression-free survival and overall survival when treated with capecitabine and oxaliplatin in people with Colorectal Neoplasms as compared to genotypes GG + AG." PTGS2 will be listed in the relationships file as "associated" with "Colorectal Neoplasms", but in reality the gene is associated with the treatment outcome for capecitabine and oxaliplatin in people who have the condition.
- ***Gene-Gene*** associations are derived from a PharmGKB pathway in which one gene influences another gene (direct connection from pathway arrows).

NOTE: "VariantLocation" refers to a genetic variant such as a SNP, indel or repeat, typically identified by the dbSNP rsID.

NOTE: In PharmGKB, "haplotype" refers to a named variant or combination of variants commonly used in the field, such as the star haplotype nomenclature for the cytochrome P450s (eg. CYP2C9*3, CYP2D6*5).

Column Headings:

- **Entity1_id**
    - Diseases, genes and drugs are designated by their PharmGKB IDs. Mappings between PharmGKB IDs and disease/gene/drug names are found in the "genes.zip", "drugs.zip" and "diseases.zip" files on the Download tab (http://www.pharmgkb.org/downloads.jsp). VariantLocation1 is typically identified by the dbSNP rsID, but may be labeled by chromosomal position. Haplotype2 is typically identified by a haplotype name, i.e. CYP2D6*4.
- **Entity1_type**
    - Disease, Drug, Gene, VariantLocation1 or Haplotype2.
- **Entity2_id**
    - Diseases, genes and drugs are designated by their PharmGKB IDs. Mappings between PharmGKB IDs and disease/gene/drug names are found in the "genes.zip", "drugs.zip" and "diseases.zip" files on the Download tab (http://www.pharmgkb.org/downloads.jsp). VariantLocation1 is typically identified by the dbSNP rsID, but may be labeled by chromosomal position. Haplotype2 is typically identified by a haplotype name, i.e. CYP2D6*4.
- **Entity2_type**
    - Disease, Drug, Gene, VariantLocation1 or Haplotype2.
- ***Evidence***
    - VIP, VariantAnnotation, ClinicalAnnotation, DosingGuideline, DrugLabel or Pathway. Comma separated list because the evidence for a relationship could come from multiple sources in PharmGKB.
- ***Association***
    - Possible values: "associated", "not associated" or "ambiguous".
        - "associated" means an association between the entities is supported by the "Evidence" and "PMIDs" columns.
        - "not associated" means that the entities were evaluated but not found have a statistically significant association based on the "PMIDs" column.
        - "ambiguous" means that some of the items in the "Evidence" and/or "PMIDs" columns support an association and others do not.
- ***PK***
    - PK stands for "Pharmacokinetic". Relationships are marked as PK if the pair of entities was found in a pharmacokinetic pathway on PharmGKB, or if the Variant Annotation or VIP was annotated with PK in some manner.
    - The absence of PK in this column does NOT necessarily mean that there is NO evidence of a pharmacokinetic relationship.
- ***PD***
    - PD stands for "Pharmacodynamic". Relationships are marked as PD if the pair of entities was found in a pharmacodynamic pathway on PharmGKB, or if the Variant Annotation or VIP was annotated with PD in some manner.
    - The absence of PD in this column does NOT necessarily mean that there is NO evidence of a pharmacodynamic relationship.
- ***PMIDs***
    - PubMed IDs that were used to support the listed relationship. Semi-colon delimited list.
